# Survival of stage II nasopharyngeal carcinoma patients with or without concurrent chemotherapy: A propensity score matching study

**DOI:** 10.1002/cam4.2785

**Published:** 2019-12-20

**Authors:** Di‐Han Liu, Xiao‐Yu Zhou, You‐Guang Pan, Si Chen, Zheng‐Hao Ye, Gang‐Dong Chen

**Affiliations:** ^1^ The Third Affiliated Hospital of Guangzhou Medical University Guangzhou P. R. China; ^2^ The First Affiliated Hospital of Jinan University Guangzhou P. R. China; ^3^ Sun Yat‐sen University Cancer Centre Guangzhou P. R. China

**Keywords:** concurrent chemotherapy, nasopharyngeal carcinoma, propensity score matching, stage II

## Abstract

**Background:**

To ascertain if concurrent chemotherapy (CCT) benefits people with stage II nasopharyngeal carcinoma (NPC) treated with two‐dimensional radiotherapy (2DRT) or intensity‐modulated radiotherapy (IMRT).

**Methods:**

A total of 4157 patients diagnosed with stage II NPC were evaluated. Patients received radiotherapy (RT) with/without CCT. Patients were divided into 2DRT and IMRT subgroups. After propensity score matching, the role of CCT was explored in these two subgroups. Overall survival (OS) was the primary endpoint and progression‐free survival (PFS), locoregional relapse‐free survival (LRFS) and distant metastasis‐free survival (DMFS) were secondary endpoints.

**Results:**

In the 2DRT subgroup, CCT addition to RT benefited cases with T1N1/T2N1 in OS, PFS and LRFS (*P* < .001, *P* = .003 and *P* = .003, respectively) significantly, but no difference was observed in patients with T2N0. DMFS were similar in the two arms. CCT was a significant protective factor for OS, PFS, and LRFS for patients with stage N1. In the IMRT subgroup, RT alone could maintain equivalent OS, PFS, LRFS and DMFS (*P* = .209, .448, .477 and .602 respectively) and cause less acute toxicity compared with concurrent chemoradiotherapy (CCRT).

**Conclusion:**

CCRT was better than 2DRT alone among patients with T1‐2N1M0 stage. CCT application for NPC patients receiving IMRT led to no survival benefit and greater toxic effects.

## Graphical abstract

In this study, we explored if concurrent chemotherapy (CCT) benefits people with stage II nasopharyngeal carcinoma (NPC) treated with two‐dimensional radiotherapy (2DRT) or intensity‐modulated radiotherapy (IMRT).

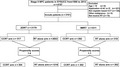

## 1 INTRODUCTION

Radiotherapy (RT) is the only curative treatment for Nasopharyngeal cancer (NPC) due to its radiosensitivity.^1^ Concurrent chemoradiotherapy (CCRT) is recommended for locoregional advanced NPC.^2^, ^3^, ^4^ Patients with early‐stage NPC are, in general, considered to have a better chance of survival. However, studies have shown that patients with stage II NPC had a relatively worse prognosis, especially those with stage T1‐T2N1 NPC.^5^ Therefore, exploring the value of CCRT for patients with stage II NPC is important.

Previously, patients treated with RT alone were reported to have significantly worse 5‐year overall survival (OS) compared with the CCRT group according to a phase‐III randomized trial studying the efficacy of CCRT for patients with stage II NPC.^6^ However, all the patients in the study were evaluated in the era of conventional two‐dimensional radiotherapy (2DRT).

With the development of technology, mathematics and computer science, intensity‐modulated radiotherapy (IMRT) has replaced 2DRT in centers where this radiation technology is available. IMRT is superior to 2DRT in terms of locoregional control of cancer and improving the quality of life of patients as a result of its spatial dose distribution in the target volume.^7^, ^8^, ^9^ Moreover, whether the outcome of stage II NPC patients in the 2DRT era applies to IMRT should be taken into serious consideration.

Zhang et al compared patients with low‐risk NPC (T1N1M0, T2N0‐1M0, T3N0M0) who underwent IMRT with/without concurrent chemotherapy (CCT).^10^ They observed no survival benefit from addition of platinum‐based CCT. However, 87 (18.0%) patients with stage III NPC were enrolled in that study. The role of CCT in different RT technologies for stage II NPC is still unknown. Therefore, we carried out this retrospective study with a large cohort and long duration of follow‐up to analyze CCRT vs RT alone among patients with stage II NPC in different eras using conventional 2DRT and IMRT.

## 2 MATERIALS AND METHODS

### 2.1 Patients

Four thousand one hundred and fifty‐seven consecutive and unselected patients diagnosed with stage II NPC from 1992 to 2012 at Sun Yat‐sen University Cancer Center were assessed. We restaged all patients according to the seventh TNM staging manual from the American Joint Committee on Cancer.^11^ Nasopharyngoscopy and magnetic resonance imaging (MRI) with contrast of the nasopharynx and neck were conducted before treatment. Contrast‐enhanced computed tomography (CT) of the chest and the abdomen were also examined. Positron emission tomography CT was applied when needed.

The exclusion criteria of this study were: (a) age < 18 years; (b) not cisplatin‐based CCT; (c) not receiving RT; (d) with other malignancies; (v)application of adjuvant chemotherapy or induction chemotherapy. The study protocol was approved by the institutional review board. Written informed consent was obtained from every patient.

### 2.2 Chemotherapy and RT

In 2002 or earlier, the main RT method was 2DRT; IMRT was conducted increasingly since the year 2003. 2DRT or IMRT was undertaken five times a week at ≈2 Gy per day. The accumulated radiation dose of a primary tumor was 66‐72 Gy. The types of RT methods and the plan of IMRT have been reported previously.^12^, ^13^, ^14^, ^15^ Cisplatin (30‐40 mg/m^2^ every week during RT) or the dose of 80‐100 mg/m^2^ for 2‐3 cycles was used in CCT.

### 2.3 Follow‐up and outcome

After treatment, the patients were subsequently followed‐up every 3 months during the first 3 years, every 6 months during the next 3 years, and then annually. Patients who were lost to follow‐up or were still alive without distant metastasis or locoregional recurrence at the end of the trial had their data censored at the date of last follow‐up. OS was the time from the date of diagnosis to death. Progression‐free survival (PFS) was defined as the interval between the date of the diagnosis and first failure or death. Locoregional relapse‐free survival (LRFS) was considered as the interval between the date of the diagnosis and the date of first local and/or regional failure. Distant metastasis‐free survival (DMFS) was defined as the interval between the date of the diagnosis and the date of distant metastasis detection.

### 2.4 Statistical analyses

Patients were divided into 2DRT and IMRT subgroups according to the RT method. We matched treatment groups using propensity scores to address the imbalance of potential confounders between the groups. The propensity score for each patient was calculated to estimate their probability using multivariable logistic regression models. The propensity‐score model included age, sex, pathologic type, T stage, N stage, diabetes mellitus, cardiovascular disease, chronic infection with hepatitis B virus, smoking, family history of NPC, and calendar periods. We then formed matched pairs between 2DRT and IMRT subgroups patients using the nearest neighbor‐matching method with a 1:4 matching protocol in the 2DRT subgroup and a 1:1 matching protocol in the IMRT subgroup, both with a caliper of 0.05. The statistical relationship between the subgroups was analyzed using the Pearson χ^2^ test or Fisher's exact test. Variables were entered into a Cox proportional hazards regression model for multivariate analyses with estimation of the corresponding hazard ratio (HR), 95% confidence interval (CI), and probability. All reported *P*‐values were two‐tailed, and *P* < .05 was considered statistically significant. Statistical analyses were performed using SPSS v21 (IBM).

## 3 RESULTS

### 3.1 Patient characteristics

**Figure 1:**
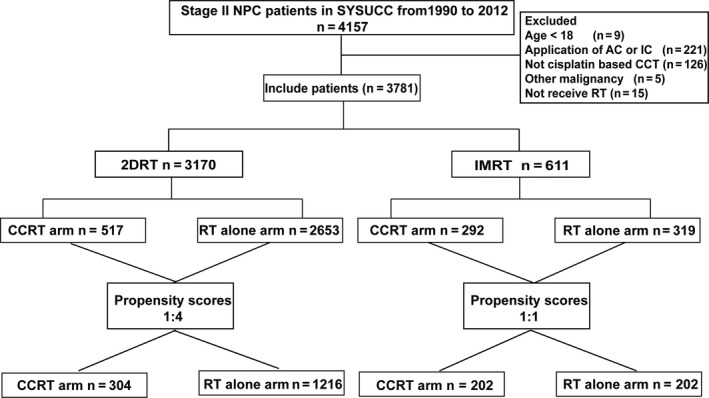
Flow chart used for patient enrollment and propensity score matching

Between January 1990 and December 2012, 4157 consecutive patients with stage II NPC received treatment at the Sun Yat‐sen University Cancer Center. Among these 4157 patients, 2986 (71.8%) were treated with RT alone and 1156 (25.6%) received CRT, among whom 935 (22.5%) were treated with CCRT and 221 (5.3%) received induction chemotherapy or adjuvant chemotherapy. Finally, 3781 (93.8%) patients were eligible for this study after exclusion (Figure 1).

In the original dataset (n = 3781), significant differences in baseline characteristics were observed between the two groups (Table S1). Then, 1520 patients treated with 2DRT (RT alone: 1216; CCRT: 304) were selected after matching by 1:4 propensity score matching (PSM) and 404 patients treated with IMRT (RT alone: 202; CCRT: 202) were selected. The baseline information of this balanced cohort is shown in Table 1. Significant differences in potential prognostic factors were not observed in the RT group or CCRT group after the matching.

**Table 1:** Baseline characteristics of patients in the well‐balanced cohort

Characteristic	2D‐RT (n = 1520)		*P*	IMRT (n = 404)		*P*
	RT (n = 1216)	CCRT (n = 304)		RT (n = 202)	CCRT (n = 202)	
Age, y			.442^a^			.486^a^
Median (range)	46 (18‐81)	45 (23‐73)		46 (18‐75)	45 (21‐73)	
≤45	590 (48.5)	155 (51.0)		98 (48.5)	105 (52.0)	
>45	626 (51.5）	149 (49.0)		104 (51.5)	97 (48.0)	
Gender			.124^a^			.165^a^
Female	354 (29.1)	75 (24.7)		71 (35.1)	58 (28.7)	
Male	862 (70.9)	229 (75.3)		131 (64.9)	144 (71.3)	
Pathological type			.939^a^			.522^a^
WHO type II	35 (2.9)	9 (3.0)		4 (2.0)	6 (3.0)	
WHO type III	1181 (97.1)	295 (97.0)		198 (98.0)	196 (97.0)	
T stage^b^, *			.877^a^			.264^a^
T1	267 (22.0)	68 (22.4)		60 (29.7)	50 (24.8)	
T2	949 (78.0)	236 (77.6)		142 (70.3)	152 (75.2)	
N stage^b^, *			.648^a^			.293^a^
N0	230 (18.9)	61 (20.1)		39 (19.3)	31 (15.3)	
N1	986 (81.1)	243 (79.9)		163 (80.7)	171 (84.7)	
Diabetes mellitus			.600^b^, *			.359^a^
No	1192 (98.0)	300 (98.7)		194 (96.0)	190 (94.1)	
Yes	24 (2.0)	4 (1.3)		8 (4.0)	12 (5.9)	
Cardiovascular disease			.536^a^			.681^b^, *
NO	1180 (97.0)	297 (97.7)		200 (99.0)	198 (98.0)	
Yes	36 (3.0)	7 (2.3)		2 (1.0)	4 (2.0)	
Chronic HBV infection			.372^a^			.368^b^, *
No	1187 (97.6)	294 (96.7)		198 (98.0)	201 (99.5)	
Yes	29 (2.4)	10 (3.3)		4 (2.0)	1 (0.5)	
Smoking			.214^a^			.087^a^
No	704 (57.9)	164 (53.9)		146 (72.3)	130 (64.4)	
Yes	512 (42.1)	140 (46.1)		56 (27.7)	72 (35.6)	
Family history of NPC			.744 a			1.000^a^
No	1080 (88.8)	272 (89.5)		178 (88.1)	1178 (88.1)	
Yes	136 (11.2)	32 (10.5)		24 (11.9)	24 (11.9)	
Calendar periods			.524 a			.473^a^
1990‐1996	470 (38.7)	120 (39.5)		—	—	
1997‐2002	228 (18.8)	58 (19.1)		—	—	
2003‐2007	383 (31.5)	85 (28.0)		48 (23.8)	42 (20.8)	
2008‐2012	135 (11.1)	41 (13.5)		154 (76.2)	160 (79.2)	

Abbreviations: HBV, hepatitis B virus; NPC, nasopharyngeal carcinoma.

a *P* values were calculated by Chi‐square test.

b *P* value calculated by correction for continuity Chi‐square test.

* According to the 7th edition of UICC/AJCC staging system.

### 3.2 Survival outcomes in the 2DRT era

**Figure 2:**
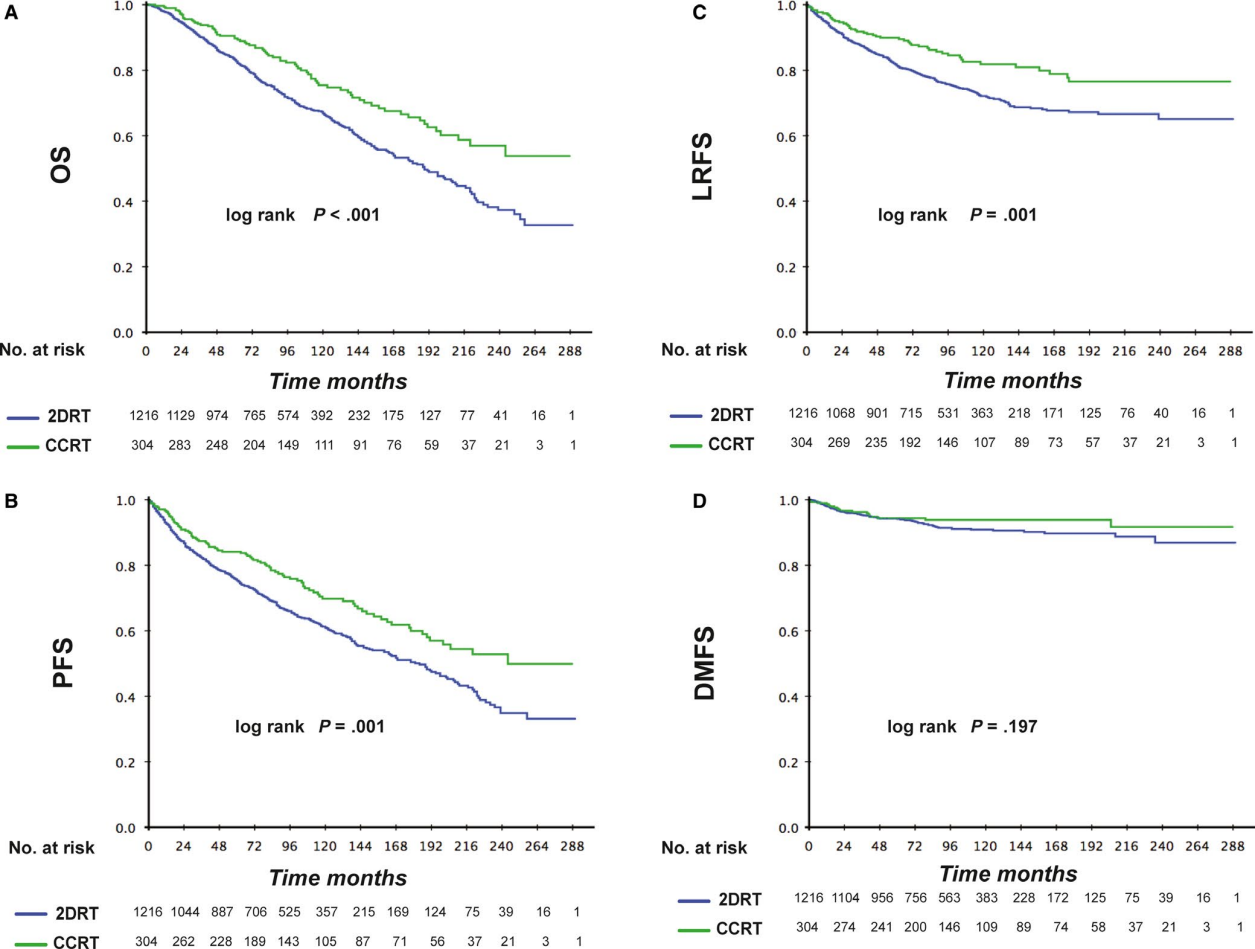
Kaplan‐Meier curves of 2DRT‐alone and CCRT subgroups of stage II NPC patients for Overall survival (A), Progression‐free survival (B), Locoregional relapse‐free survival (C) and Distant metastasis‐free survival (D)

The median duration of follow‐up for the cohort of patients treated with 2DRT was 93 (range 2‐290) months. Differences in OS, PFS and LRFS between the RT‐alone and CCRT group were significant except for DMFS (*P* < .001, *P* = .003, *P* = .003, and *P* = .197, respectively) (Figure 2A‐D). Table 2 shows OS, PFS, LRFS and DMFS at 3, 5, 7, 10, 15 and 20 years in the two groups. OS was higher for patients in the CCRT group than for patients in the 2DRT group at each time point. Similar results were found for PFS and LRFS, but there was no significant difference for DMFS.

**Table 2:** Survival outcomes for the patients with stage II NPC in the different arms

	2D‐RT (n = 1520)		IMRT (n = 404)	
	RT (n = 1216)	CCRT (n = 304)	RT (n = 202)	CCRT (n = 202)
	%, 95% CI	%, 95% CI	%, 95% CI	%, 95% CI
OS				
Rate at 3 y	90.3 (88.5‐92.1)	93.8 (91.1‐96.5)	98.4 (96.6‐100)	97.5 (95.3‐99.7)
Rate at 5 y	83.2 (81.0‐85.4)	89.7 (86.2‐93.2)	98.4 (96.6‐100)	95.9 (92.2‐99.6)
Rate at 7 y	75.1 (72.6‐77.6)	83.9 (79.4‐88.4)	94.8 (87.5‐102)	90.4 (82.2‐98.6)
Rate at 10 y	66.8 (63.9‐69.7)	74.7 (68.8‐80.6)		
Rate at 15 y	51.5 (47.4‐55.6)	65.6 (58.3‐72.9)		
Rate at 20 y	37.3 (31.6‐42.9)	53.8 (43.4‐64.2)		
PFS				
Rate at 3 y	82.3 (80.1‐84.5)	87.7 (83.9‐91.4)	92.6 (88.9‐96.3)	90.8 (86.7‐94.9)
Rate at 5 y	75.3 (72.8‐77.8)	83.7 (79.4‐88.0)	91.8 (87.7‐95.9)	87.5 (82.0‐92.9)
Rate at 7 y	68.9 (66.2‐71.6)	78.4 (73.5‐83.3)	85.8 (76.6‐95.0)	84.8 (77.4‐92.2)
Rate at 10 y	60.9 (57.8‐64.0)	69.8 (63.7‐75.9)		
Rate at 15 y	50.1 (46.2‐54.0)	60.0 (52.7‐67.3)		
Rate at 20 y	34.9 (29.2‐40.6)	49.9 (40.1‐59.7)		
LRFS				
Rate at 3 y	87.7 (85.7‐89.7)	91.8 (88.7‐94.9)	95.2 (92.1‐98.3)	94.5 (91.2‐97.8)
Rate at 5 y	81.8 (79.6‐83.9)	89.5 (85.9‐93.0)	95.2 (92.1‐98.3)	92.6 (88.5‐96.7)
Rate at 7 y	77.7 (75.2‐80.2)	86.2 (82.1‐90.3)	95.2 (92.1‐98.3)	92.6 (88.5‐96.7)
Rate at 10 y	71.9 (68.9‐74.8)	81.8 (76.7‐86.9)		
Rate at 15 y	67.2 (63.7‐70.7)	76.5 (70.0‐82.9)		
Rate at 20 y	65.1 (60.4‐69.8)			
DMFS				
Rate at 3 y	95.3 (94.1‐96.5)	95.9 (93.5‐98.3)	97.2 (94.8‐99.6)	95.2 (92.1‐98.3)
Rate at 5 y	94.1 (92.7‐95.5)	94.4 (91.7‐97.1)	94.1 (89.2‐99.0)	93.7 (89.6‐97.8)
Rate at 7 y	92.0 (90.2‐93.8)	93.9 (90.9‐96.8)	91.8 (85.3‐98.3)	92.6 (88.5‐96.7)
Rate at 10 y	90.6 (88.6‐92.6)	91.7 (86.6‐96.8)		
Rate at 15 y	88.7 (85.8‐91.6)			
Rate at 20 y	86.9 (82.4‐91.4)			

Abbreviations: CI, confidence interval; DMFS, distant metastasis‐free survival; LRFS, locoregional relapse free survival; NPC, nasopharyngeal carcinoma; OS, overall survival; PFS, progression‐free survival.

In the multivariate analysis, the following factors were evaluated: age, sex, T stage, N stage, smoking, family history of NPC, and type of treatment. CCRT was associated with significantly better OS, PFS and LRFS than the RT‐alone group (OS: HR, 0.623; 95% CI, 0.487‐0.796; *P* < .001; PFS: 0.685; 0.548‐0.856; 0.001; LRFS: 0.616; 0.455‐0.834; 0.002) but was not an independent prognostic factor for DMFS. Age and N stage were also prognostic factors for OS, PFS and LRFS (Table 3).

**Table 3:** Multivariable analysis of prognostic factors for OS, PFS, LRFS and DMFS of the patients with stage II NPC

Characteristic	2D‐RT(n = 1520)		IMRT(n = 404)	
	Hazard ratio (95% CI)	*P*	Hazard ratio (95% CI)	*P*
Overall survival				
Age (y)	2.009 (1.673‐2.413)	<.001	4.273 (0.921‐19.817)	.064
Gender	1.263 (0.998‐1.597)	.052	0.439 (0.042‐4.628)	.493
T stage	1.113 (0.895‐1.384)	.338	0.604 (0.163‐2.240)	.451
N stage	1.677 (1.304‐2.157)	<0001	1.576 (0.308‐8.055)	.585
Smoking	1.130 (0.921‐1.386)	.241	9.119 (1.179‐70.565)	.034
Family history of NPC	0.853 (0.633‐1.149)	.294	0.442 (0.052‐3.738)	.454
Type of treatment	0.623 (0.487‐0.796)	<.001	0.537 (0.159‐1.816)	.318
Progression‐free survival				
Age (y)	1.837 (1.550‐2.177)	<.001	0.878 (0.462‐1.670)	.692
Gender	1.214 (0.977‐1.509)	.080	0.818 (0.333‐2.011)	.661
T stage	1.145 (0.933‐1.404)	.195	1.872 (0.808‐4.334)	.143
N stage	1.551 (1.232‐1.952)	<.001	1.942 (0.739‐5.105)	.178
Smoking	1.096 (0.906‐1.326)	.344	2.959 (1.371‐6.385)	.006
Family history of NPC	0.849 (0.643‐1.122)	.250	0.912 (0.320‐2.600)	.864
Type of treatment	0.685 (0.548‐0.856)	.001	0.838 (0.440‐1.595)	.590
Locoregional relapse‐free survival				
Age (y)	1.724 (1.387‐2.143)	<.001	0.969 (0.408‐2.300)	.943
Gender	1.174 (0.895‐1.539)	.248	0.686 (0.196‐2.406)	.557
T stage	1.208 (0.925‐1.578)	.165	2.796 (0.811‐9.640)	.104
N stage	1.575 (1.169‐2.122)	.003	3.049 (0.693‐13.415)	.140
Smoking	0.964 (0.755‐1.231)	.770	4.030 (1.368‐11.872)	.011
Family history of NPC	0.758 (0.521‐1.103)	.148	0.809 (0.185‐3.531)	.778
Type of treatment	0.616 (0.455‐0.834)	.002	0.812 (0.341‐1.935)	.638
Distant metastasis‐free survival				
Age (y)	1.403 (0.963‐2.043)	.078	0.346 (0.122‐0.980)	.046
Gender	1.299 (0.794‐2.125)	.297	1.072 (0.301‐3.822)	.914
T stage	1.257 (0.777‐2.032)	.351	1.636 (0.529‐5.062)	.393
N stage	1.162 (0.719‐1.880)	.540	4.186 (0.545‐32.175)	.169
Smoking	1.046 (0.685‐1.599)	.834	2.682 (0.931‐7.725)	.068
Family history of NPC	1.155 (0.660‐2.023)	.614	1.673 (0.473‐5.923)	.425
Type of treatment	0.719 (0.434‐1.191)	.200	0.861 (0.337‐2.197)	.754

Note

A Cox proportional hazards regression model was used to detect variables one by one without adjustment. All variables were transformed into categorical variables. HRs were calculated for Age (y) (>45 vs ≤45); Gender (M vs F); T stage (II vs I); N stage (I vs 0); Smoking (Yes vs No); Family history of NPC (Yes vs No); Type of treatment (CRT vs RT).

Abbreviation: CI, confidence interval.

### 3.3 Subgroup analyses in the 2DRT era

**Figure 3:**
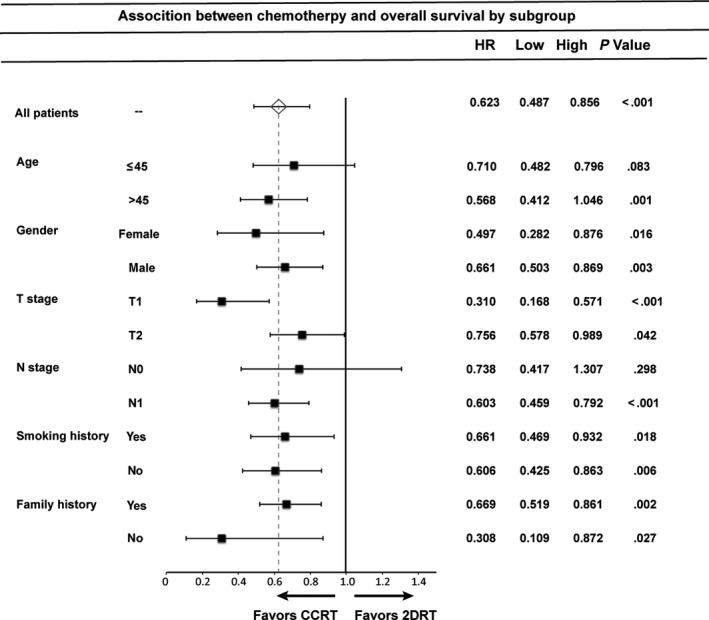
Forest plot of the association between chemotherapy and overall survival by subgroup. Legend: Multivariate hazard ratios (HR) displayed were adjusted for the selected factors. Lower limit of the 95% confidence interval (Low). Upper limit of the 95% confidence interval (High)

Patients at different N stages exhibited different risks of metastasis and prevalence of treatment failure. Thus, we divided patients according to the N stage (N0 and N1) and compared the prognostic impact of adding chemotherapy in these two groups. Patients with stage‐N0 disease showed no significant difference in clinical outcome between the different treatments (Figure S1), whereas CCRT was associated with better OS, PFS and LRFS than RT alone in the N1 subgroup (Figure S2). Multivariate survival analysis also showed that CCRT treatment was an independent prognostic factor for OS, PFS and LRFS in patients with N1 disease (*P* < .001, .002 and .003, respectively) (Table 4), but did not show survival benefit in the N0 subgroup. Figure 3 shows the forest plot of the association between treatment type and overall survival by subgroup. Multivariate hazard ratios (HR) were adjusted for the selected factors (age, gender, T stage N stage, smoking history and family history) excluding the stratification covariates.

**Table 4:** Multivariable analysis of prognostic factors for OS, PFS, LRFS and DMFS of the patients with stage II NPC

	N0(n = 291)		N1(n = 1229)	
Characteristic	Hazard ratio (95% CI)	*P*	Hazard ratio (95% CI)	*P*
Overall survival				
Age (y)	2.098 (1.274‐3.454)	.004	2.006 (1.646‐2.444)	<.001
Gender	1.360 (0.722‐2.562)	.342	1.250 (0.970‐1.611)	.084
T stage	—	—	1.110 (0.892‐1.380)	.350
Smoking	1.140 (0.677‐1.920)	.621	1.129 (0.904‐1.410)	.283
Family history of NPC	0.932 (0.428‐2.032)	.860	0.841 (0.609‐1.162)	.294
Type of treatment	0.738 (0.417‐1.307)	.298	0.603 (0.459‐0.792)	<.001
Progression‐free survival				
Age (y)	1.846 (1.184‐2.880)	.007	1.845 (1.534‐2.219)	<.001
Gender	1.251 (0.717‐2.182)	.431	1.205 (0.952‐1.527)	.121
T stage	—	—	1.143 (0.932‐1.403)	.199
Smoking	1.022 (0.635‐1.646)	.928	1.109 (0.901‐1.365)	.330
Family history of NPC	0.858 (0.415‐1.774)	.679	0.845 (0.625‐1.142)	.272
Type of treatment	0.732 (0.430‐1.245)	.249	0.677 (0.530‐0.866)	.002
Locoregional relapse‐free survival				
Age (y)	1.750 (0.983‐3.114)	.057	1.728 (1.365‐2.186)	<.001
Gender	1.351 (0.642‐2.845)	.428	1.148 (0.857‐1.537)	.355
T stage	—	—	1.204 (0.922‐1.572)	.174
Smoking	1.033 (0.561‐1.903)	.917	0.952 (0.729‐1.242)	.715
Family history of NPC	1.135 (0.484‐2.659)	.771	0.698 (0.460‐1.061)	.092
Type of treatment	0.669 (0.326‐1.373)	.273	0.605 (0.433‐0.845)	.003
Distant metastasis‐free survival				
Age (y)	2.371 (0.911‐6.169)	.077	1.248 (0.825‐1.889)	.294
Gender	0.804 (0.280‐2.309)	.686	1.493 (0.852‐2.614)	.161
T stage	—	—	1.264 (0.782‐2.045)	.339
Smoking	0.852 (0.304‐2.391)	.761	1.075 (0.673‐1.715)	.763
Family history of NPC	0.427 (0.057‐3.183)	.406	1.326 (0.736‐2.388)	.347
Type of treatment	0.829 (0.278‐2.478)	.738	0.699 (0.395‐1.236)	.218

Note

A Cox proportional hazards regression model was used to detect variables one by one without adjustment. All variables were transformed into categorical variables. HRs were calculated for Age (y) (>45 vs ≤45); Gender (M vs F); T stage (II vs I); Smoking (Yes vs No); Family history of NPC (Yes vs No); Type of treatment (CRT vs RT).

Abbreviation: CI, confidence interval.

### 3.4 Survival outcomes in the IMRT era

**Figure 4:**
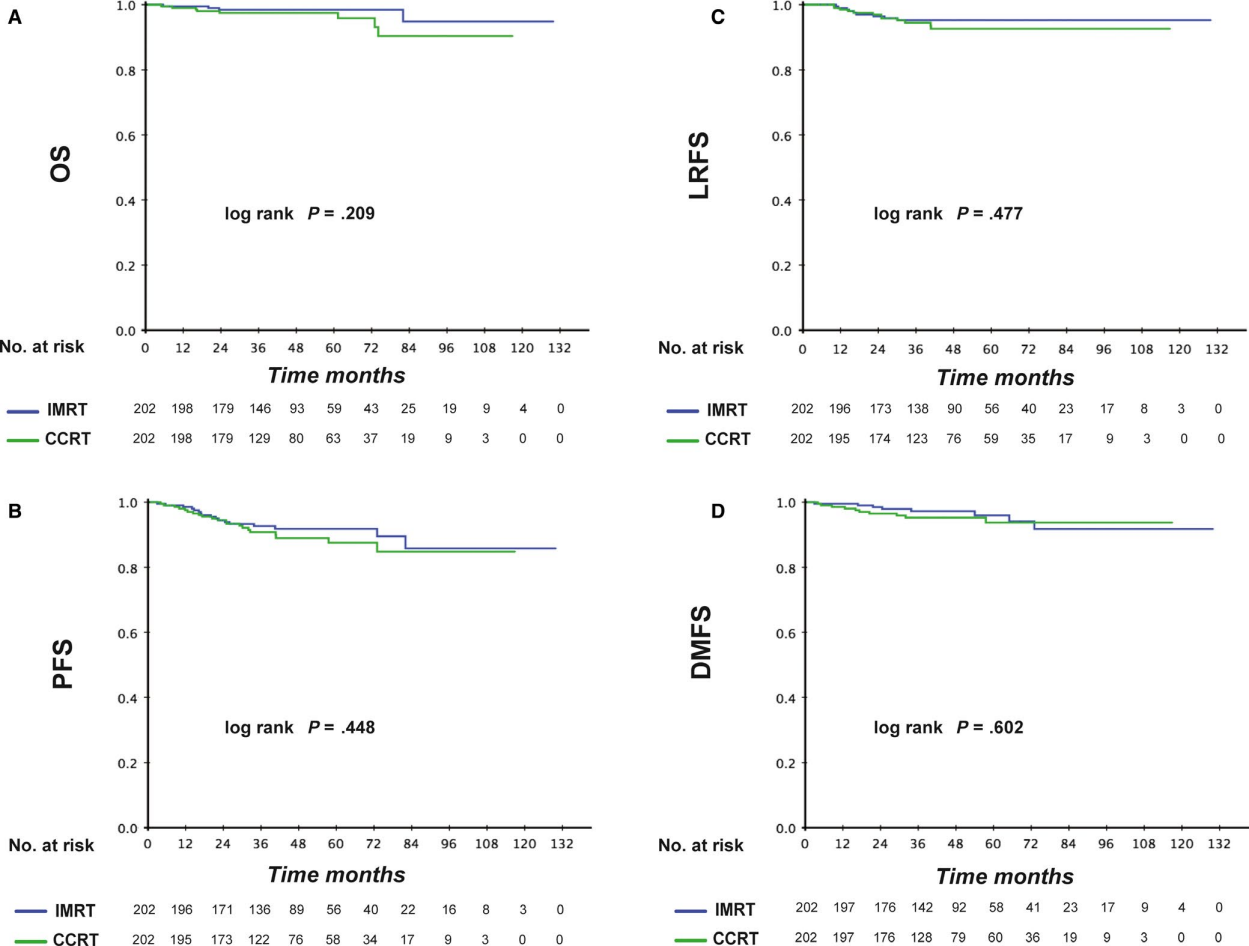
Kaplan‐Meier curves of IMRT alone and CCRT subgroups of stage‐N1 NPC patients for Overall survival (A), Progression‐free survival (B), Locoregional relapse‐free survival (C) and Distant metastasis‐free survival (D)

The median duration of follow‐up for patients treated with IMRT was 44 (range, 5‐130) months. Differences in OS, DFS, LRFS and DMFS between the RT‐alone group and CCRT were not significant (*P* > .2 for all) (Figure 4). The details of OS, PFS, LRFS and DMFS in the two groups are illustrated in Table 2. OS at 3 and 5 years was similar in the two treatment groups (95.9% to 98.4%). Similar results were found for PFS, LRFS and DMFS. It seemed that the addition of chemotherapy did not extend the lives of NPC patients. In addition, after adjustment for various factors, CCRT was not established as an independent prognostic factor for all types of survival (Table 3). However, smoking was a prognostic factor for OS, PFS and LRFS in the IMRT group.

### 3.5 Acute toxicity in the IMRT era

During IMRT, complete hematology results were available for 341 patients and we analyzed the toxic effect in these patients. Patients in the CCRT group experienced significantly more hematologic toxicities than patients in the RT‐alone group: leucocytopenia (grade 1‐2:58.1% vs 32.2%; grade 3‐4:10.2% vs 1.7%; *P* < .001), neutropenia (38.9% vs 10.3%; 8.4% vs 0.6%; <0.001), anemia (28.1% vs 4.6%; 0.6% vs 0.6%; <0.001), and thrombocytopenia (15.6% vs 2.3%; 2.4% vs 0.6%; <0.001) (Table 5). Besides, CCT significantly increased the prevalence of grade 1‐2 hepatoxicity (37.7% vs 17.2%). No significant differences among the treatment arms were observed in terms of nephrotoxicity.

**Table 5:** Acute toxicities between the two arms of patients treated with RT or CCRT

Adverse event (toxicity grade)	RT (n = 174)			CCRT (n = 167)			*P*
	0(%)	1‐2(%)	3‐4(%)	0 (%)	1‐2(%)	3‐4(%)	
Leukocytopenia	115 (66.1)	56 (32.2)	3 (1.7)	53 (31.7)	97 (58.1)	17 (10.2）	<.001^a^
Neutropenia	155 (89.1)	18 (10.3)	1 (0.6)	88 (52.7)	65 (38.9)	14 (8.4）	<.001^a^
Anemia	165 (94.8)	8 (4.6)	1 (0.6)	119 (71.3)	47 (28.1)	1 (0.6)	<.001^b^
Thrombocytopenia	169 (97.1)	4 (2.3)	1 (0.6)	137 (82.0)	26 (15.6)	4 (2.4)	<.001^b^
Hepatoxicity	143 (82.2)	30 (17.2)	1 (0.6)	103 (61.7)	63 (37.7)	1 (0.6)	<.001^b^
Nephrotoxicity	161 (92.5)	13 (7.5）	0 (0.0)	148 (95.2)	18 (4.2)	1 (0.6)	.331^b^

Abbreviations: CCRT, concurrent chemoradiotherapy; RT, radiotherapy.

a *P* values were calculated by Chi‐square test.

b *P* value calculated with Fisher's exact test.

## 4 DISCUSSION

CCRT was established as a standard treatment protocol in patients with locoregional advanced NPC because of the high risk of locoregional recurrence and distant metastasis.^2^, ^3^, ^4^ However, whether application of chemotherapy can improve the survival of patients with stage II NPC is not known. Some studies have shown that IMRT can prolong the survival of NPC patients with early‐stage or advanced NPC.^5^, ^16^, ^17^

We reviewed the results of 1924 stage II NPC patients treated with RT with or without CCT at the Sun Yat‐sen University Cancer Center and divided patients into two subgroups according to the RT technology employed (2DRT and IMRT).

In the 2DRT era, a phase‐III randomized study demonstrated that CCT improved the survival of patients with stage II NPC significantly^6^; OS at 5 years in the CCRT group and RT group was 70.3% (95% CI: 63.4‐77.3%) and 58.6% (50.9‐66.2%), respectively. Patients receiving CCT also showed longer PFS and DMFS compared with patients treated with RT alone. Thus, addition of CCT in the RT period among cases with stage II NPC seems reasonable. However, only 230 patients were involved in the study and only 26 patients died during a median follow‐up of 60 months.

In addition, Xu *et al* retrospectively compared 2DRT alone with 2DRT plus CCT in 392 patients with T2N1M0 NPC, and showed no difference in OS despite an improvement in LRFS.^18^ Here, we evaluated 1520 patients with stage II NPC treated with 2DRT with a longer median follow‐up (93 months). All the potential prognostic factors were balanced using PSM (1:4) to mimic randomized trials. We found that application of chemotherapy benefited patients significantly in terms of OS, LRFS and PFS, whereas DMFS was comparable between the two arms. These data supported the view that CCT helps to control local disease and achieve long‐term survival in stage II NPC. This result could have been because, in 2DRT, the therapeutic dose cannot cover all the tumor volume. Hence, chemotherapy may have an important role in “salvage treatment” to kill tumor cells out of the target volume and further improve the LRFS and PFS.

Several studies have shown that patients with stage II NPC with T1‐2N1 achieve worse clinical outcomes and may benefit from aggressive therapy.^5^, ^19^ According to the different tumor burden and prevalence of treatment failure, we divided all patients into two subgroups according to N stage and investigated the role of chemotherapy in these two subgroups. Interestingly, subgroup analyses showed that the CCRT arm had significantly better PFS, OS and LRFS than the 2DRT arm in patients with N1 stage. However, no significant difference was found in T2N0 patients. Our findings identified optimal candidates for CCT among patients with stage II NPC treated with 2DRT and direct individualized treatment.

In the IMRT era, several studies have explored the effect of CCT in patients with stage II NPC. Tham et al evaluated 107 patients with stage IIb NPC and found no significant difference in survival between patients who underwent or did not undergo CCT.^20^ In a meta‐analysis, Cheng et al demonstrated that IMRT alone could achieve equivalent OS, LRFS and DMFS compared with CRT with fewer toxic effects (*P* = .14, 0.06, and 0.89, respectively).^21^ Conversely, Kang et al observed that concurrent treatment with 5‐fluorouracil and cisplatin improved PFS and LRFS at 5 years significantly in patients with stage II NPC.^22^ In our study, CCRT failed to show benefit in all survival endpoints among a large, propensity score matched cohort. Besides, patients in the CCRT group tolerated more serious toxic effects such as leucopenia and neutropenia. Thus, IMRT alone was superior to CCRT based on therapeutic and toxic effects. This phenomenon could be due to three main reasons.

First, with the development of RT technology, IMRT has enabled “tailoring” of the dose distribution, which can improve locoregional control significantly.^7^, ^12^, ^23^ Thus, the risk of recurrence could be controlled by IMRT alone and the treatment effect of chemotherapy was weakened. Second, the high‐grade liver dysfunction and renal impairment caused by chemotherapy may have obscured the survival benefit of chemotherapy, resulting in unfavorable outcomes compared with IMRT alone. Third, although we matched all patients to balanced potential prognostic factors between the two arms, the illness severity differed among each prognostic factor. For example, N1 stage denoted patients with unilateral metastasis in cervical lymph node(s) ≤6 cm in greatest dimension above the border of cricoid cartilage. The size of the metastatic lymph node meant that many patients had different illness severities. Besides, it is reasonable that chemotherapy was applied for patients with a serious illness. The clinician's choice of treatment plan may have reduced the benefits of CCT to a nonsignificant effect.

This retrospective study had the largest sample of cases with stage II NPC. PSM and multivariate analysis were used to increase the reliability of the results, which was the major strength of this study. However, our study had several limitations. First, there were no data on late toxicities and we failed to incorporate some important recognized prognostic factors such as EBV DNA. Second, our data were retrospectively taken from a single institution, the pathological type of 98.0% patients was type III and there were clinical and pathological differences between the patients in CCRT group and IMRT group. Further multicenter research into the prognostic effects of CCT is warranted. Finally, although we selected patients with cisplatin‐based CCT, the heterogeneity of chemotherapy doses was an inevitable bias.

In the treatment of patients with stage II NPC, CCRT was better than 2DRT for OS, PFS and DMFS, especially for patients with N1 stage. IMRT was superior to CCRT with no survival difference and lower prevalence of toxic effects.

## CONFLICT OF INTEREST

The authors claim no conflict of interest.

## AUTHORSHIP

Conception and design were done by Di‐Han Liu, You‐Guang Pan, and Gang‐Dong Chen. Di‐Han Liu, Zheng‐Hao Ye and Si Chen collected and assembled the data. Data analyses and interpretation were done by Di‐Han Liu, Xiao‐Yu Zhou and You‐Guang Pan. The manuscript was written and approved by all authors.

## Data Availability

The datasets used in the study are available from the corresponding author on reasonable request. The data are not publicly available due to privacy or ethical restrictions.
