# Supplementary figures and images for: Survival of stage II nasopharyngeal carcinoma patients with or without concurrent chemotherapy: A propensity score matching study
Source: Cancer Med. 2019 Dec 20;9(4):1287–97. doi: 10.1002/cam4.2785 (PMC7013074; doi:10.1002/cam4.2785)

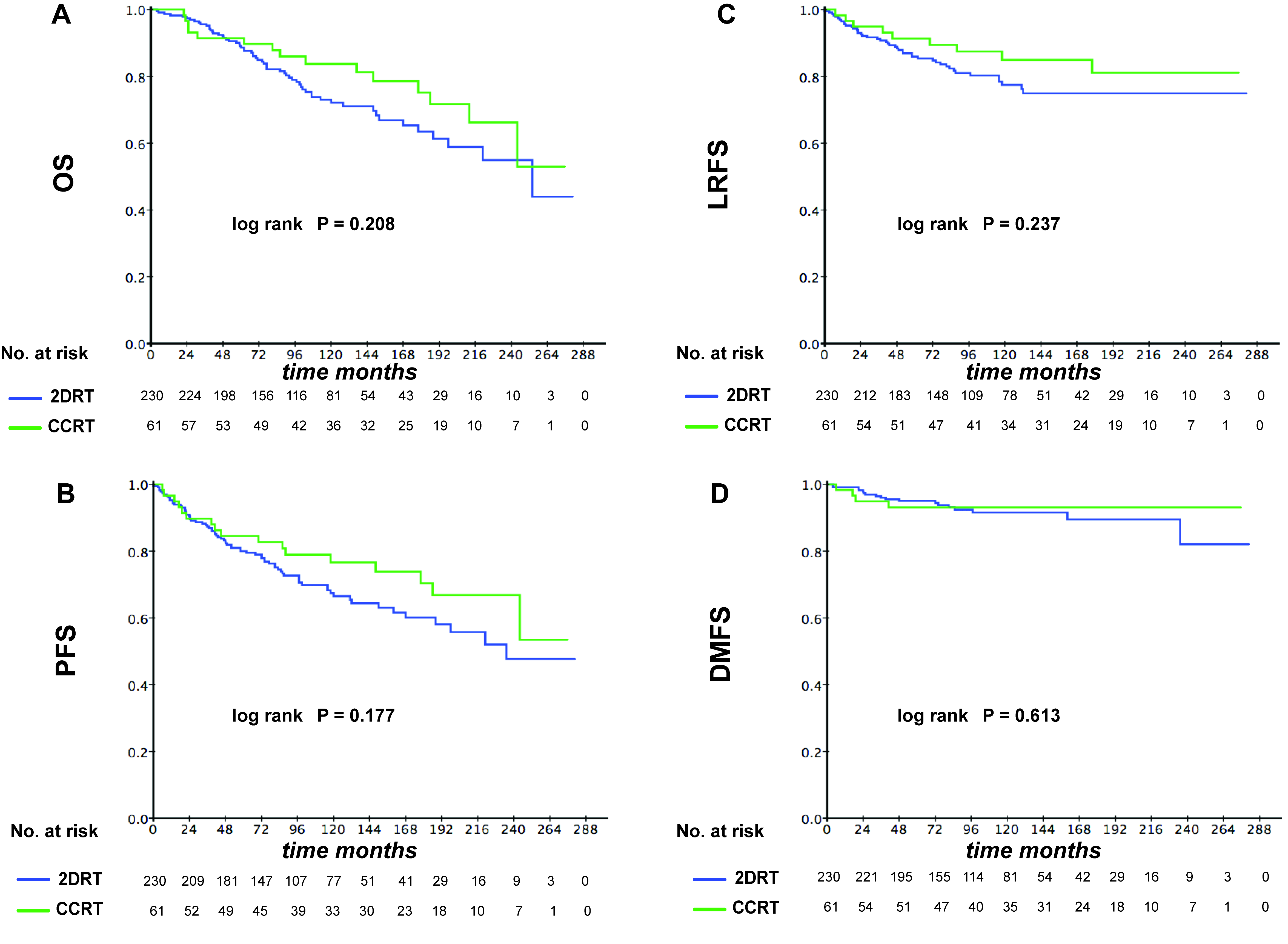

Supplement: Supplementary file 1 [file CAM4-9-1287-s001.tif]

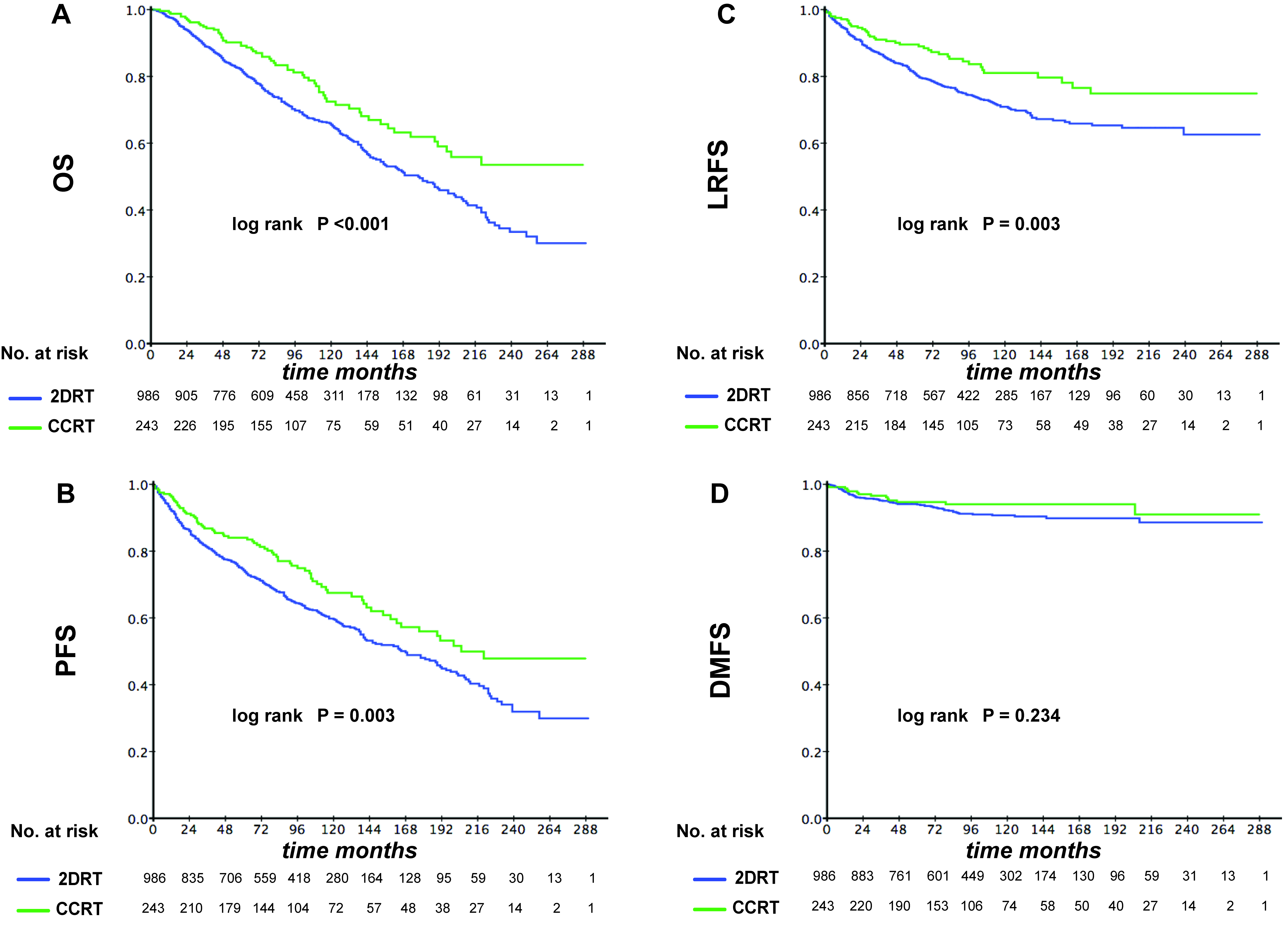

Supplement: Supplementary file 2 [file CAM4-9-1287-s002.tif]
