# Supplementary material for: Survival of stage II nasopharyngeal carcinoma patients with or without concurrent chemotherapy: A propensity score matching study
Source: Cancer Med. 2019 Dec 20;9(4):1287–97. doi: 10.1002/cam4.2785 (PMC7013074; doi:10.1002/cam4.2785)
Supplement: Supplementary file 3 [file CAM4-9-1287-s003.docx]

**Supplement Table**

Table S1. Baseline characteristics of patients in the whole cohort

|  | 2D-RT(n=3170) | |  | IMRT(n=611) | | | |  | |  |  |  |
| --- | --- | --- | --- | --- | --- | --- | --- | --- | --- | --- | --- | --- |
| **Characteristic** | RT (n=2653) | CCRT(n=517) | *P* | RT (n=319) | | CCRT (n=292) | | *P* | |  |  |  |
| **Age, y** |  |  | 0.147^a^ |  | |  | | 0.380^a^ | |  |  |  |
| Median(range) | 46(18-81) | 45 (23-73) |  | 46(18-75) | | 45(21-73) | |  | |  |  |  |
| ≤45 | 1247(47.0) | 261(50.5) |  | 158(49.5) | | 155(53.1) | |  | |  |  |  |
| >45 | 1406(53.0） | 256(49.5) |  | 161(50.5) | | 137(46.9) | |  | |  |  |  |
| **Gender** |  |  | 0.109^a^ |  | |  | | 0.797^a^ | |  |  |  |
| Female | 759(28.6) | 130(25.1) |  | 97(30.4) | | 86(29.5) | |  | |  |  |  |
| Male | 1894(59.7) | 387(74.9) |  | 222(69.6) | | 206(70.5) | |  | |  |  |  |
| **Pathological type** | |  | 0.035^c^ |  | |  | | 0.575^a^ | |  |  |  |
| WHO type I | 1(0.0) | 1(0.2) |  | - | | - | |  | |  |  |  |
| WHO type II | 50(1.9) | 19(3.7) |  | 10(3.1) | | 7(2.4) | |  | |  |  |  |
| WHO type III | 2592(98.1) | 496(96.1) |  | 308(96.9) | | 285(97.6) | |  | |  |  |  |
| **T stage*** |  |  | 0.318^a^ |  | |  | | 0.098^a^ | |  |  |  |
| T1 | 430(16.2) | 93(18.0) |  | 60(18.8) | | 71(24.3) | |  | |  |  |  |
| T2 | 2223(83.8) | 424(82.0) |  | 259(81.2) | | 221(75.7) | |  | |  |  |  |
| **N stage*** |  | <0.001^a^ | |  | | <0.001^a^ | | | |  |  |  |
| N0 | 1044(39.4) | 112(21.7) |  | 156(48.9) | | 43(14.7) | |  | |  |  |  |
| N1 | 1609(60.6) | 405(78.3) |  | 163(51.1) | | 249(85.3) | |  | |  |  |  |
| **Diabetes mellitus** | |  | 0.595^a^ |  | |  | | 0.260^a^ | |  |  |  |
| No | 2615(98.6) | 508(98.3) |  | 303(95.0) | | 271(92.8) | |  | |  |  |  |
| Yes | 38(1.4) | 9(1.7) |  | 16(5.0) | | 21(7.2) | |  | |  |  |  |
| **Cardiovascular disease** | |  | 0.785^a^ |  | |  | | 0.840^b^ | |  |  |  |
| NO | 2586(97.5) | 505(97.7) |  | 314(98.4) | | 288(98.6) | |  | |  |  |  |
| Yes | 67(2.5) | 12(2.3) |  | 5(1.6) | | 4(1.4) | |  | |  |  |  |
| **Chronic HBV infection** | | 0.538^a^ | | 0.052^b^ | | | | | |  |  |  |
| No | 2607(98.3) | 506(97.9) |  | 313(98.1) | | 292(100) | |  | |  |  |  |
| Yes | 46(1.7) | 11(2.1) |  | 6 (1.9) | | 0(0.0) | |  | |  |  |  |
| **Smoking** | | 0.936^a^ | |  | | 0.478^a^ | | | |  |  |  |
| No | 1503(56.8) | 292(56.6) |  | 213(66.8) | | 187(64.0) | |  | |  |  |  |
| Yes | 1144(43.2) | 224(43.4) |  | 106(33.2) | | 105(36.0) | |  | |  |  |  |
| **Family history of NPC** | | 0.564 ^a^ | |  | | 0.865 ^a^ | | | |  |  |  |
| No | 2373(89.4) | 458(88.6) |  | 276(86.5) | | 254(87.0) | |  | |  |  |  |
| Yes | 280(10.6) | 59(11.4) |  | 43(13.5) | | 38(13.0) | |  | |  |  |  |
| **Calendar periods** | |  | <0.001 ^a^ | |  | |  | | <0.001 ^a^ | |  |  |
| 1990-1996 | 1084(40.9) | 137(26.5) |  | - | | - | |  | |  |  |  |
| 1997-2002 | 909(34.3) | 69(13.3) |  | - | | - | |  | |  |  |  |
| 2003-2007 | 489(18.4) | 185(35.8) |  | 98(30.7) | | 42(14.4) | |  | |  |  |  |
| 2008-2012 | 171(6.4) | 126(24.4) |  | 221(69.3) | | 250(85.6) | |  | |  |  |  |

Abbreviations: HBV, hepatitis B virus; NPC, nasopharyngeal carcinoma.

^a^P values were calculated by Chi-square test. ^b^P value calculated by correction for continuity Chi-square test.

^*^According to the 7th edition of UICC/AJCC staging system
